# TREM2/β-catenin attenuates NLRP3 inflammasome-mediated macrophage pyroptosis to promote bacterial clearance of pyogenic bacteria

**DOI:** 10.1038/s41419-022-05193-x

**Published:** 2022-09-06

**Authors:** Yi Wang, Can Cao, Yanting Zhu, Huifeng Fan, Qiaojuan Liu, Yiting Liu, Kang Chen, Yongjian Wu, Siping Liang, Meiyu Li, Lexi Li, Xi Liu, Yuanqing Zhang, Chenglin Wu, Gen Lu, Minhao Wu

**Affiliations:** 1 Department of Laboratory Medicine, The Second Affiliated Hospital of Guangzhou University of Chinese Medicine, Guangzhou, 510120 China; 2 Program of Infection and Immunity, Affiliated Guangzhou Women and Children’s Hospital, Zhongshan School of Medicine, Sun Yat-sen University, Guangzhou, China; 3 Key Laboratory of Tropical Diseases Control, Ministry of Education, Sun Yat-sen University, Guangzhou, 510080 China; 4 School of Pharmaceutical Sciences, Sun Yat-sen University, Guangzhou, 510006 China; 5 Organ Transplant Center, The First Affiliated Hospital, Sun Yat-sen University, Guangzhou, China; 6 Guangdong Engineering & Technology Research Center for Disease-Model Animals, Sun Yat-sen University, Guangzhou, 510006 China

**Keywords:** Immune cell death, Infection, Inflammation

## Abstract

Triggering receptors expressed on myeloid cells 2 (TREM2) is considered a protective factor to protect host from bacterial infection, while how it elicits this role is unclear. In the present study, we demonstrate that deficiency of triggering receptors expressed on myeloid cells 2 (TREM2) significantly enhanced macrophage pyroptosis induced by four common pyogenic bacteria including *Staphylococcus aureus*, *Pseudomonas aeruginosa*, *Streptococcus pneumoniae*, and *Escherichia coli*. TREM2 deficiency also decreased bacterial killing ratio of macrophage, while Caspase-1 or GSDMD inhibition promoted macrophage-mediated clearance to these bacteria. Further study demonstrated that the effect of TREM2 on macrophage pyroptosis and bacterial eradication mainly dependents on the activated status of NLRP3 inflammasome. Moreover, as the key downstream of TREM2, β-catenin phosphorylated at Ser675 by TREM2 signal and accumulated in nucleus and cytoplasm. β-catenin mediated the effect of TREM2 on NLRP3 inflammasome and macrophage pyroptosis by reducing NLRP3 expression, and inhibiting inflammasome complex assembly by interacting with ASC. Collectively, TREM2/β-catenin inhibits NLRP3 inflammasome to regulate macrophage pyroptosis, and enhances macrophage-mediated pyogenic bacterial clearance.

## Introduction

Pyogenic bacteria including *Staphylococcus aureus*, *Pseudomonas aeruginosa*, *Streptococcus pneumoniae*, and *Escherichia coli* are major opportunistic human pathogens that often lead to a variety of pyogenic infections, such as community-acquired or hospital-acquired pneumonia [1], bacterial keratitis [2], skin or soft tissue infection, urinary tract infection and bacteremia [3, 4]. The emergence of increasing multiple antibiotic resistance of these bacteria in past decades has made bacterial infection a significant global health concern [5]. As a result of studying the relationship between host and pathogens, we realized host immune status are critical to infection control and can be considered promising therapeutic targets [6].

Innate immunity is the first-line of host antibacterial defense. As the major phagocytes against invading pathogens, macrophages and neutrophils contribute critically to bacterial clearance and inflammatory regulation [7]. After recognizing various bacterial components via pattern recognition receptors (PRRs) [8], phagocytes engulf invading pathogens and kill them intracellularly. Triggering receptors expressed on myeloid cells 2 (TREM2) is a broadly expressed PRR on macrophages, dendritic cells, and microglia cells [9, 10], which be considered an important therapeutic target for neurodegenerative disease, infectious disease and tumor immunotherapy [11, 12]. TREM2 conducts signal through DNAX-activating protein of 12 kDa (DAP12), negatively modulating inflammatory response during bacterial infection [13, 14]. It was reported that TREM2 can bind to bacterial anionic molecules, such as lipopolysaccharide (LPS), dextran sulfates and cell debris, while its downstream signals vary in response to distinct stimulation[15]. Our previous in vivo studies showed that TREM2 protects *Pseudomonas aeruginosa*-infected mouse corneas by attenuating tissue inflammation, cells pyroptosis and bacterial grown [14, 16]. However, the mechanism of TREM2 regulating immune cells pyroptosis and bacterial clearance remains unclear.

Activation of PRRs in phagocytes also induces pyroptosis, a pro-inflammatory cell death associated with inflammasome. NLRP3 is an critical intracellular PRR involved in canonical inflammasome signaling, which can be activated by various PAMPs or DAMPs and then assembles with ASC and pro-Caspase-1, leading to ASC oligomerization and pro-Caspase-1 cleavage [17]. Activated Caspase-1 then processes pro-IL-1β and GSDMD to IL-1β and N-GSDMD, respectively. N-GSDMD forms pores on cell membrane, resulting in pyroptotic cell death and release of inflammatory intracellular components such as LDH and IL-1β [18]. Inflammasome-triggered immune cells pyroptosis plays a vital role in host antibacterial response and inflammatory injury, while its role in regulating defense against bacterial infection remains controversial [19]. Studies reported that macrophage pyroptosis is beneficial to the elimination of intracellular bacterium including *Salmonella typhimurium* [20], *Legionella pneumophila*, and *Francisella tularensis* [21]. However, inflammasome activation and pyroptosis ensued in immune cells also been found play deleterious roles in multiple pyogenic bacteria caused infection by amplifying tissue inflammation and attenuating pathogens clearance [16, 22, 23]. Therefore, the role and underlying mechanism of immune cells pyroptosis in against distinct bacterial infection needs further research.

Colonna’s group reported that deficiency of TREM2 impairs the stabilization of β-catenin during the process of osteoclast differentiation [24], and both DAP12 and β-catenin are involved in macrophage colony-stimulated factor (M-CSF)-induced macrophage proliferation [25]. β-catenin is an evolutionarily conserved molecule, which plays an important role in cell proliferation, differentiation, and apoptosis [26]. Our previous in vivo study demonstrated that β-catenin promotes host resistance against *Pseudomonas aeruginosa* corneal infection [27]. Nonetheless, whether TREM2 and β-catenin are synergistic in regulating macrophages pyroptosis and bacterial clearance needs further investigation.

In the present study, we explored the mechanism of TREM2 in regulating macrophage pyroptosis and defense against pyogenic bacteria. We found that TREM2 suppresses NLRP3 inflammasome activation and macrophage pyroptosis to promote pyogenic bacteria clearance. Furthermore, TREM2 promotes the β-catenin stability by regulating its phosphorylation at Ser675, and stabilized β-catenin suppresses NLRP3 inflammasome by inhibiting *NLRP3* transcription and inflammasome complex assembly. Our findings provide a better understanding for the interaction between host immunity and pathogens, which may explore a potential therapeutic target for the treatment of pyogenic bacterial infection.

## Materials and methods

### Materials and reagents

Ac-YVAD-CMK (caspase-1 inhibitor) and LPS derived from *Pseudomonas aeruginosa* were purchased from Sigma-Aldrich (St. Louis, MO, USA). Nigericin and flagellin derived from *Pseudomonas aeruginosa* were purchased from InvivoGen (San Diego, CA, USA). Primary antibodies: anti-β-catenin (WB) and anti-β-actin from Sigma-Aldrich; anti-TREM2(WB, IP) from Abcam (Cambridge, MA, USA); anti-β-catenin (IP), anti-phosphorylated β-catenin (S33/37, S552, S675), anti-phosphorylated Akt and anti-phosphorylated GSK-3β from Cell Signaling Test (CST; Beverly, MA, USA); anti-Caspase-1 (p10), anti-Lamin B from Santa Cruz Biotechnology (Santa Cruz, CA, USA); anti-Caspase-1 (p20), anti-NLRP3, anti-NLRC4 and anti-ASC from Adipogen (San Diego, CA, USA). Detail catalog information of antibodies and reagents provide in Supplementary Table 1.

### Bacterial strains

*Pseudomonas aeruginosa* strain (19660), *Staphylococcus aureus* strain (25923), *Streptococcus pneumoniae* strain (49136), *Salmonella Typhimurium* (13311), and *Escherichia coli* strain (25922) was purchased from American Type Culture Collection (ATCC; Manassas, VA, USA). *Pseudomonas aeruginosa*, *Staphylococcus aureus*, *Escherichia coli*, *Salmonella Typhimurium* were grown on LB plates medium at 37 °C. *Streptococcus pneumoniae* were grown at Columbia blood plate medium at 37 °C in a humidified incubator with 5% CO_2_.

### Experimental animals

Six-week-old C57BL/6 (B6) female mice were purchased from Sun Yat-sen University Animal Supply Center. TREM2^−/−^ (KO) female mice in B6 background were generously provided by Marco Colonna (Washington University School of Medicine) and bred in Sun Yat-sen University Animal Supply Center. Caspase-1^−/−^ (Casp1 KO) female mice in B6 background were purchased from Jackson Laboratory (Bar Harbor, ME, USA). All animals were housed in Specified Pathogen Free environment. All animal experiments complied with the French Government animal experiment regulations and ARRIVE guidelines, and were performed in accordance with the National Institutes of Health Guide for the Care and Use of Laboratory Animals, with the approval of the Scientific Investigation Board of Sun Yat-sen University (Guangzhou, Guangdong, China).

### Isolation and culture of bone marrow-derived macrophages

Mouse bone marrow-derived macrophages (BMDMs) were isolated and differentiated as reported previously. In brief, WT, TREM2 KO or Casp1 KO mice were sacrificed by Spinal dislocation and the bone marrow in femurs was flushed out and cultured in DMEM containing 10% FBS, 1% penicillin–streptomycin, 1% L-glutamine (all from Invitrogen, Carlsbad, CA, USA) and 30% (vol/vol) conditioned medium of L-929 cells (mouse fibroblast cell line), as a source of M-CSF. BMDMs were obtained as a homogeneous population of adherent cells after 7 days culture. The BMDM purity was routinely >95% as assessed by flow cytometry.

### Cell culture and transient transfection

iBMDMs were kindly provided by Feng Shao (National Institute of Biological Sciences, Beijing). Murine macrophage-like RAW264.7 cells (TIB-71) were obtained from ATCC. These cells were cultured in DMEM medium supplemented with 10% fetal bovine serum (FBS), 1% penicillin–streptomycin, and 1% L-glutamine. Cells were incubated at 37 °C in a humidified incubator with 5% CO_2_. According to the manual of Lipofectamine™ 2000 (Invitrogen), iBMDMs or RAW264.7 were transiently transfected with pcDNA3.1 vector or plasmids encoding wild-type (WT) or mutant (S675A, S552A) β-catenin, or specific small interfering RNA against TREM2 (siTREM2), β-catenin (siβ-cat) or GSDMD (siGSDMD) vs. scrambled control siRNA (siNC). Each siRNA was mixed by two siRNAs that target different sequences of the same specific gene. All siRNAs were synthesized by Ruibo Biotechnology (Guangzhou, Guangdong, China). Flagellin transfection was performed according to the manual of DOTAP Liposomal Transfect Reagent (Sigma-Aldrich).

### Lentivirus transduction and establishment of iBMDM cells with stable β-catenin expression

A lentiviral plasmid encoding β-catenin with 45′ serine, 41′ threonine, 37′ serine, and 33′ serine mutation was purchased from Addgene (Seattle, WA, USA). Production of lentivirus vectors was carried out as described previously [27]. Briefly, HEK293T cells (ATCC, Manassas, VA, USA) were transfected with 2.5 μg lentiviral plasmid, 0.8 μg VSV-G enveloping plasmid and 1.7 μg pCMVDR8.2 packaging plasmid. After 48 h post-transfection, cell supernatants were collected and centrifuged at 4000 × *g* for 5 min to discard cell fragments. Viral titer was measured by the HIV p24 Antigen ELISA kit (Zeptometrix, Buffalo, NY). Next, iBMDM cells were infected by β-catenin-lentivirus or vector-lentivirus at a multiple of infection (MOI) of 10 for 6 h in the presence of 8 mg/ml polybrene (Sigma). Infected cells were sorted by limiting dilution assay till single cell clones of β-catenin-iBMDM (β-cat-iBM) or control vector-iBMDM (vec-iBM) cells that expressing mCherry fluorescence protein were observed by fluorescence microscopy (Olympus IX53, Olympus, Japan) and then collected for further culture and assays.

### Lactate dehydrogenase (LDH) cytotoxicity assay and ELISA

iBMDMs or BMDMs were treated as indicated above, cell supernatants and lysate were collected to analyze cell death by LDH cytotoxicity assay, using CytoTox 96 Non-Radioactive Cytotoxicity Assay kit (Promega, Madison, WI, USA). ELISA was conducted to analyze IL-1β secretion in cell supernatants, using specific ELISA kits from BD Biosciences. Each sample was assayed in duplicate. Results are shown as the means ± SEM of three independent experiments.

### Real-time PCR

Total RNA was isolated from cell pellets using TRIzol (Invitrogen, Carlsbad, CA, USA) according to the manufacturer’s instruction, and quantitated using a NanoDrop 2000C Spectrophotometers (Thermo Scientific, West Palm Beach, FL, USA). 1 μg of total RNA was reverse-transcribed to produce cDNA by using RevertAid First Strand cDNA synthesis kit (Thermo Fisher Scientific, Waltham, MA, USA). Then the cDNA was amplified using SYBR green master mix (TaKaRa, Mountain View, CA, USA) following the manufacturer’s protocol. Quantitative Real-time PCR reactions were performed using a CFX96 Real-Time PCR System (Bio-Rad, Hercules, CA, USA). Relative mRNA levels were calculated after normalization to the level of β-actin.

### Western-blot

Cells were washed three times with ice-cold PBS and then lysed in lysis buffer containing 1 mM phenylmethylsulfonyl fluoride, 1% (v/v) protease inhibitor cocktail, 1% (v/v) phosphorylase inhibitor cocktail, and 1 mM dithiothreitol (all from Sigma, St. Louis, MO, USA). Nuclear and cytoplasmic protein was isolated by using a Nuclear and Cytoplasmic Extraction Kit (Thermo Scientific, West Palm Beach, FL, USA). Cell supernatants were mixed with methanol and chloroform and centrifuged in 4 °C to precipitate the protein, and then protein pellets were dissolved in lysis buffer. Equal amounts of cell lysates were resolved by SDS-PAGE and then transferred to polyvinylidene difluoride membranes. The membranes were blocked in 5% Bovine Serum Albumin PBST (pH 7.4 PBS, 0.5% Tween20) at room temperature for 1 h and incubated at 4 °C overnight with the respective primary antibodies, followed by a second incubation at room temperature for 1 h with appropriate HRP-conjugated secondary antibodies. Finally, blots on the membranes were visualized with Plus-ECL (KeyGEN, Nanjing, China) according to the manufacturer’s protocol.

### ASC oligomerization assay

Cells were lysed in 300 μl Triton Buffer [50 mM Tris-HCl (pH 7.5), 150 mM NaCl, 0.5% Triton X-100, and EDTA-free protease inhibitor cocktail]. 30μl of the lysates used as input control. The rest of cell lysates were centrifuged at 6000 × *g* at 4 °C for 15 min and the pellets were washed twice and resuspended with Triton Buffer, followed by cross-linking with 2 mM disuccinimidyl suberate (DSS) for 30 min at room temperature. Then the pellets were collected by centrifugation, and dissolved in SDS loading buffer. Precipitated pellets and soluble lysates were simultaneously immunoblotted using anti-ASC Ab.

### Co-immunoprecipitation assay

BMDMs from B6 mice or iBMDM cell were challenged with LPS (3 h) or LPS plus ATP for 1 h, and then cells were collected and lysed with Pierce™IP lysis buffer (Thermo Fisher Scientific). Subsequently, cell lysates were incubated with Antibody of ASC or NLRP3, vs. their respective isotype- matched IgG control, at 4 °C overnight and then with Protein G agarose beads (Thermo Fisher Scientific) for additional 4 h. The agarose beads were collected by centrifugation and then washed four times with IP washing buffer. Proteins were eluted in reducing sample buffer and analyzed by immunoblot.

### Flow cytometry evaluation of cell death

PI staining kit (KeyGen Biotech, Nanjing, China) was used to stain cells according to the manufacturer’s instructions. After bacteria challenge, cells were washed thrice with binding buffer, and then 5 × 10^5^ cells were incubated with 500 μl binding buffer containing 5 μl PI for 5 min. Finally, cells were collected and analyzed using a Beckman Coulter EPICS XL/MCL flow cytometer (Beckman Coulter Inc, Fullerton, CA, USA).

Intracellular bacterial killing assay. Intracellular bacterial killing efficiency was assessed using plate count method as described previously [28]. Briefly, after indicated treatments, BMDMs or iBMDMs were stimulated with LPS (100 ng/ml) for 12 h, followed by *Pseudomonas aeruginosa*, *Staphylococcus aureus*, *Streptococcus pneumoniae* and Escherichia coli infection at an MOI of 20. After an hour infection, cells were washed thrice with cold PBS and cultured for 30 min in complete medium containing 200 μg/ml gentamicin to remove extracellular bacteria. After that, cells in one of the duplicate wells were lysed for calculation of internalized bacteria [CFU (1 h)]. Cells in the other duplicate well were incubated in complete medium for another 1 h. After cell lysis and bacterial culture, the number of survived intracellular bacteria [CFU (2 h)] was calculated. Killing efficiency of the intracellular bacteria was calculated using the following equation: intracellular bacterial killing = [CFU (1 h) − CFU (2 h)]/CFU (1 h) × 100%.

### Statistical analysis

The differences between two groups or among three or more groups were analyzed by using an unpaired two-tailed Student’s *t* test, or one-way analysis of variance (one-way ANOVA) with Bonferroni’s posttest, respectively. Differences were considered statistically significant when the *P* value was <0.05.

## Results

### TREM2 inhibits pyogenic bacteria-induced macrophage pyroptosis

**Fig. 1:**
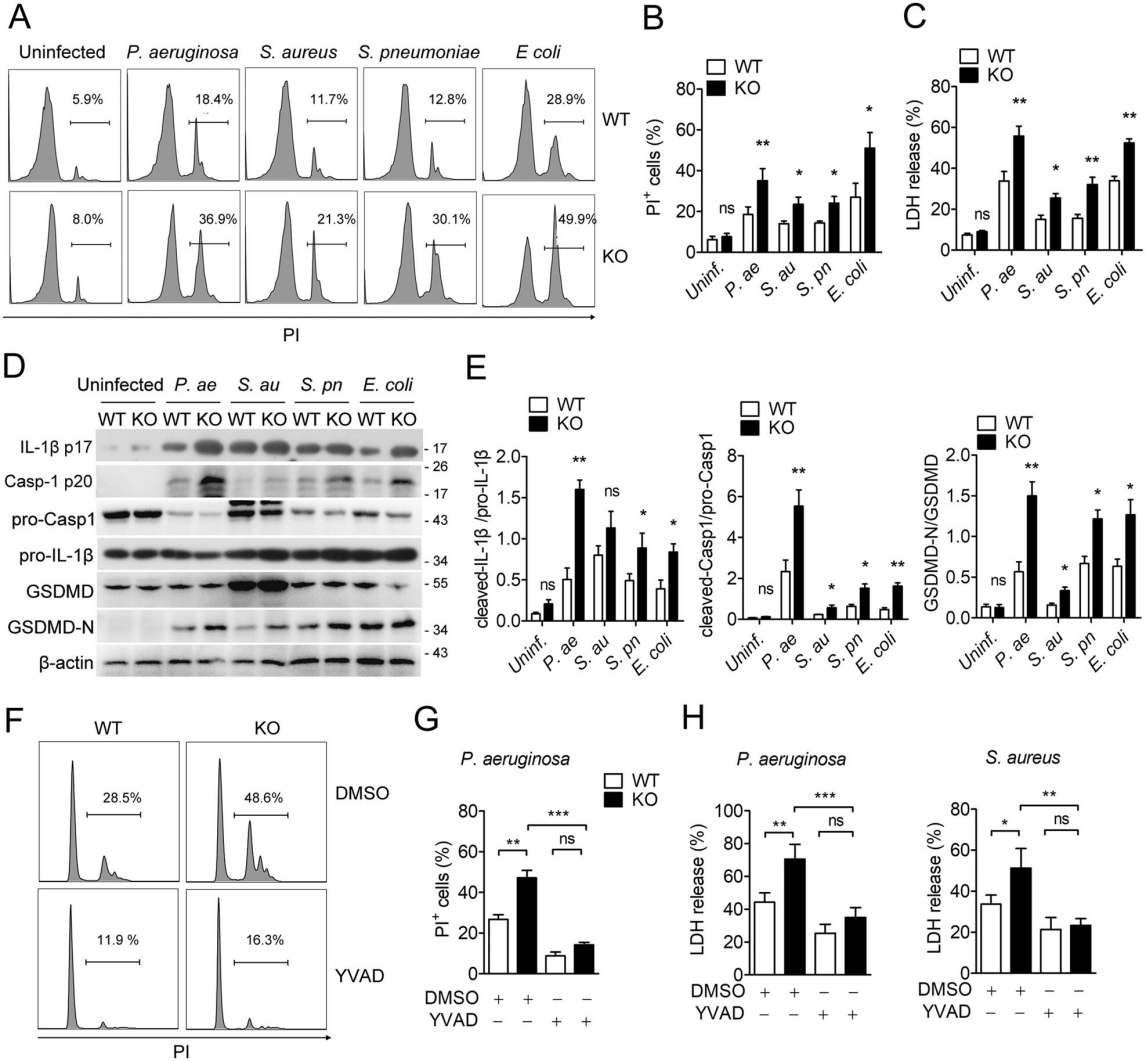
TREM2 inhibits pyogenic bacteria-induced macrophage pyroptosis. **A**–**E** BMDMs isolated from wildtype (WT) or TREM2 knockout (KO) B6 mice were stimulated with LPS (100 ng/ml) for 12 h, and then infected with *Pseudomonas aeruginosa*, *Staphylococcus aureus*, *Streptococcus pneumoniae*, *Escherichia coli* at an MOI of 10 for 3 h. **F**–**H** LPS primed WT or KO BMDMs were pretreated with vehicle control (DMSO) or Ac-YVAD-CMK (40 μM) for 45 min, followed by *Pseudomonas aeruginosa* or *Staphylococcus aureus* infection. **A**, **F** Cell death was analyzed by flow cytometry with PI staining and representative figures were shown. **B**, **G** Quantification of dead cells (PI^+^). **D** Protein levels of pro-Caspase-1, cleaved-Caspase-1, pro-IL-1β, cleaved-IL-1β, and GSDMD in cell lysates or supernatants of each group were detected by western blot. **E** Relative densities of indicated blots. **C**, **H** Cytotoxicity was detected by LDH release assay. Data are representative of at least three experiments. Error bars represent the mean ± SEM. *P < 0.05; **P < 0.01; ***P < 0.001. ns no significance.

To investigate the effect of TREM2 in pyogenic bacteria-induced macrophage death, in vitro cultured primary WT or TREM2 knockout (KO) mouse BMDMs were infected with pyogenic bacteria including *Pseudomonas aeruginosa*, *Staphylococcus aureus*, *Streptococcus pneumoniae*, Escherichia coli, respectively, and then analyzed by flow cytometry with PI staining and cytotoxicity assay. The fraction of dead cells (PI^+^) was significantly higher in KO than WT BMDMs, indicating that deficiency of TREM2 promotes macrophage death after pyogenic bacterial infection (Fig. 1A, B). Cytotoxicity assay confirmed that TREM2 deficiency enhances infection-leaded cell death as the release of LDH in KO cells was significantly higher that WT cells (Fig. 1C). Western-blot data suggested that TREM2 deficiency enhanced the cleavage of Caspase-1, IL-1β and GSDMD in BMDMs after infected with distinct pyogenic bacterium (Fig. 1D, E). Moreover, treatment with caspase-1 specific inhibitor YVAD restored the PI^+^ cell fraction (Fig. 1F, G) and LDH release (Fig. 1H) that promoted by TREM knockout in BMDMs after infection. These data together indicated that TREM2 inhibits macrophage pyroptosis induced by pyogenic bacterial infection.

### TREM2 promoted pyogenic bacteria clearance by regulating macrophage pyroptosis

**Fig. 2:**
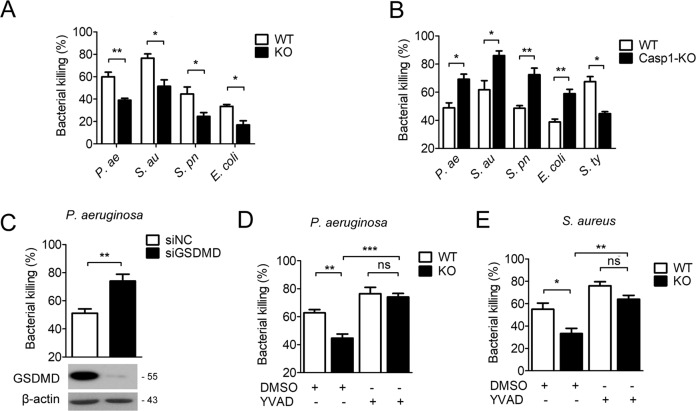
Pyroptosis suppresses macrophage-mediated pyogenic bacteria eradication. **A**, **B** TREM2 KO (KO) or Caspase-1-KO vs. WT BMDMs were infected with *Pseudomonas aeruginosa*, *Staphylococcus aureus*, *Streptococcus pneumoniae*, Escherichia coli (MOI = 20). **C** BMDMs were transiently transfected with siGSDMD vs. siNC, followed by *Pseudomonas aeruginosa* infection (MOI = 20). **D**, **E** TREM2 KO vs. WT BMDMs were pretreated with Ac-YVAD-CMK (40 μM) or DMSO, followed by *Pseudomonas aeruginosa* or *Staphylococcus aureus* infection (MOI = 20). The bacterial killing efficiency was assessed by bacterial killing assay based on plate count. **C** Protein levels of GSDMD were detected by western blot. Data are shown as the mean ± SEM of three individual experiments. *P < 0.05; **P < 0.01; ***P < 0.001. ns no significance.

To investigate the regulation of TREM2 on macrophage-mediated clearance of pyogenic bacteria, we infected WT and TREM2-KO BMDMs with *Pseudomonas aeruginosa*, *Staphylococcus aureus*, *Streptococcus pneumoniae* or Escherichia coli, and then bacterial clearance in each group were assessed by bacterial killing assay with plate count. The results showed that knockout of TREM2 in BMDMs suppressed macrophage-mediated clearance to these four pyogenic bacteria (Fig. 2A). Then we investigated the effect of pyroptosis on the bactericidal ability of macrophage by infecting WT or Caspase-1 knockout BMDMs with the four pyogenic bacteria, or a typical intracellular bacterium, *Salmonella typhimurium*. The results of bacterial killing assay showed that Caspase-1 knockout promoted the clearance of *Pseudomonas aeruginosa*, *Staphylococcus aureus*, *Streptococcus pneumoniae* or Escherichia coli, while attenuated that of *Salmonella typhimurium* (Fig. 2B). In addition, knockdown of GSDMD in BMDMs also promoted *Pseudomonas aeruginosa* killing (Fig. 2C). These results indicated that Caspase-1/GSDMD-mediated pyroptosis is detrimental to the clearance of pyogenic bacteria by macrophage.

To further assess the relationship between macrophage pyroptosis and TREM2-mediated anti-pyogenic bacteria immune defense, TREM2-KO and WT BMDMs were treated with Caspase-1 specific inhibitor and then infected with *Pseudomonas aeruginosa* or *Staphylococcus aureus*. The results of bacterial killing assay showed that knockout of TREM2 in BMDMs suppressed bacterial clearance, while blockage of Caspase-1 restored the bactericidal activity of macrophage (Fig. 2D, E). Together, these data demonstrate that TREM2 promotes pyogenic bacterial clearance by suppressing macrophage pyroptosis.

### TREM2 suppresses NLRP3 inflammasome activation upon pyogenic infection

**Fig. 3:**
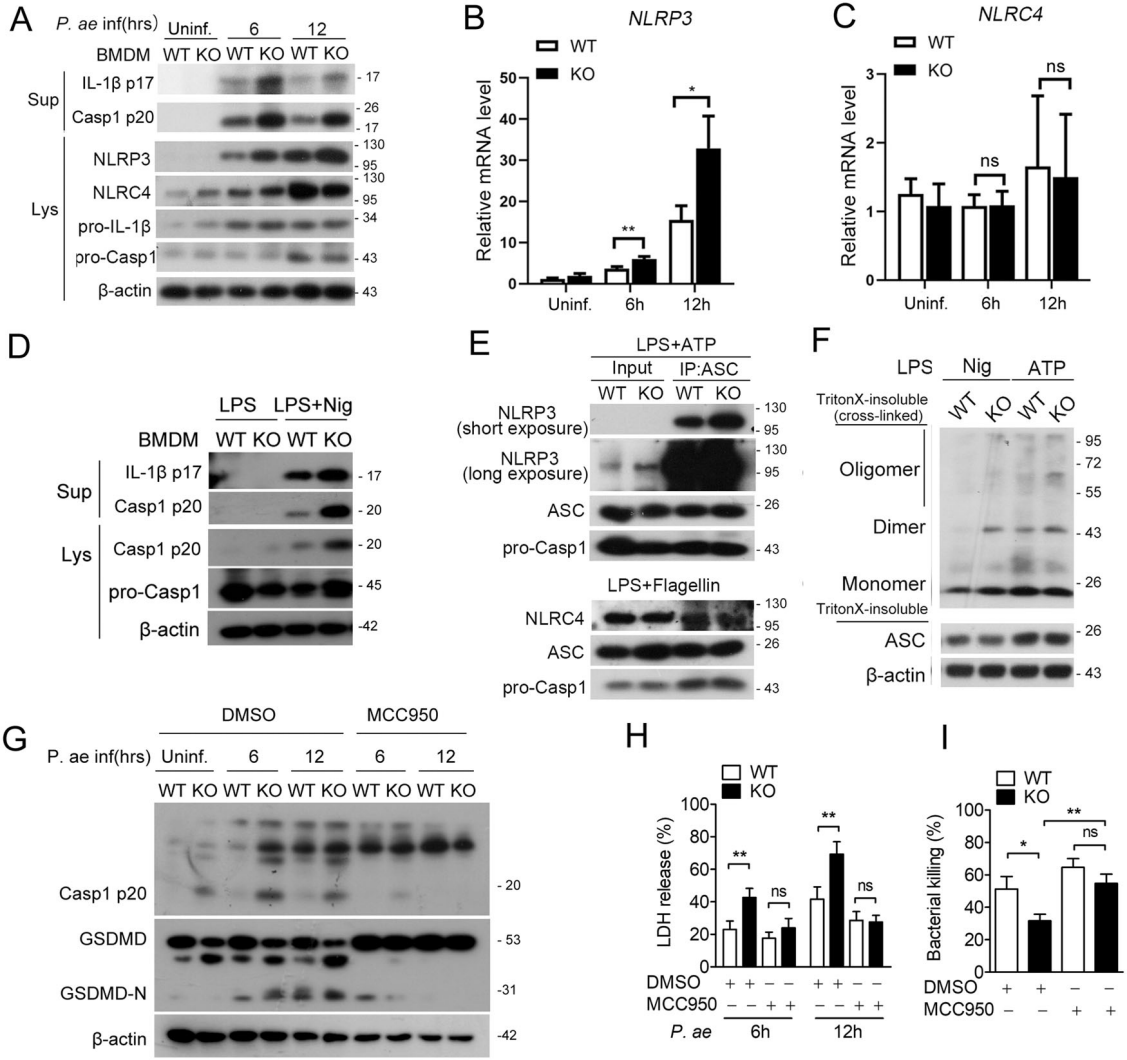
TREM2 suppresses NLRP3 inflammasome activation. **A**–**C** WT and TREM2-KO BMDMs were infected by *Pseudomonas aeruginosa* (MOI = 1) for indicated times. **D** WT and TREM2-KO BMDMs were treated with LPS or LPS plus Nigericin (10 μM) for 1 h. **E** NLRP3-ASC or NLRC4-ASC association was analyzed by immunoprecipitation in WT vs. TREM2-KO BMDMs stimulated with LPS (100 ng/ml, 3 h) plus ATP (4 mM, 1 h). **F** WT vs. TREM2-KO BMDMs were stimulated with LPS plus ATP (4 mM, 1 h), or LPS plus nigericin (10 μM, 1 h), and then cell lysates were tested by ASC oligomerization assay. **G**, **H** LPS primed WT or KO BMDMs were pretreated with vehicle control (DMSO) or MCC950 (100 nM), followed by *Pseudomonas aeruginosa* infection for indicated times. **I** WT or KO BMDMs were pretreated with vehicle control (DMSO) or MCC950 (100 nM), followed by *Pseudomonas aeruginosa* infection (MOI = 20). The bacterial killing efficiency was assessed by bacterial killing assay based on plate count. **A**, **D**, **E**, **F**, **G** Protein levels of indicated molecules in cell lysates or supernatants of each group were detected by western blot. **B**, **C** mRNA levels of NLRP3 and NLRC4 were detected by real-time PCR. **H** Cytotoxicity was detected by LDH release assay. Data are representative of at least three experiments. Error bars represent the mean ± SEM. *P < 0.05; **P < 0.01. ns no significance.

Since pyroptosis is often associated with inflammasome activation, we further tested the role of TREM2 on the activation of NLRP3 and NLRC4 inflammasome, which have been reported as the major inflammasomes induced by pyogenic bacterial infection. Interestingly, TREM2 knockout increased the expression of NLRP3 at mRNA and protein level after *Pseudomonas aeruginosa* infection while had no effect on NLRC4 (Fig. 3A–C). Besides, deficiency of TREM2 accelerated Nigericin induced Caspase‐1 and IL‐1β cleavage and release (Fig. 3D). Thus, we hypothesized that TREM2 regulating macrophage pyroptosis might mainly dependent on NLRP3 inflammasome. Then we further tested the effect of TREM2 on the assembly stage of the NLRP3 inflammasome complex. Here, Co-IP analysis showed that NLRP3-ASC interaction that response to LPS plus ATP treatment was enhanced in TREM2-KO BMDMs compared to WT control, while NLRC4-ASC interaction that response to Flagellin was not changed (Fig. 3E). In accordance with this, we also found that TREM2 knockout promoted Nigericin or ATP triggered ASC oligomerization (Fig. 3F).

To confirm whether TREM2-elicited regulation on macrophage pyroptosis and bacterial clearance were mainly associated with the function of NLRP3 inflammasome, we pretreated BMDMs were with MCC950, a NLRP3 inflammasome specific inhibitor, before *Pseudomonas aeruginosa* infection. Immunoblot data exhibited that TREM2 deficiency promoted Caspase-1 and IL-1β cleavage and release, which was abrogated by MCC950 (Fig. 3G). Consistently, cytotoxicity assay data show that MCC950 abrogated the increase of LDH release from TREM2-KO macrophages (Fig. 3H). Moreover, MCC950 treatment restored the attenuated bacterial clearance activity in TREM2 deficiency macrophages (Fig. 3I). Taken together, these results revealed that TREM2 suppresses NLRP3 inflammasome and cells pyroptosis by inhibiting NLRP3 protein expression and complex assembly.

### TREM2 promotes β-catenin stabilization in macrophages

**Fig. 4:**
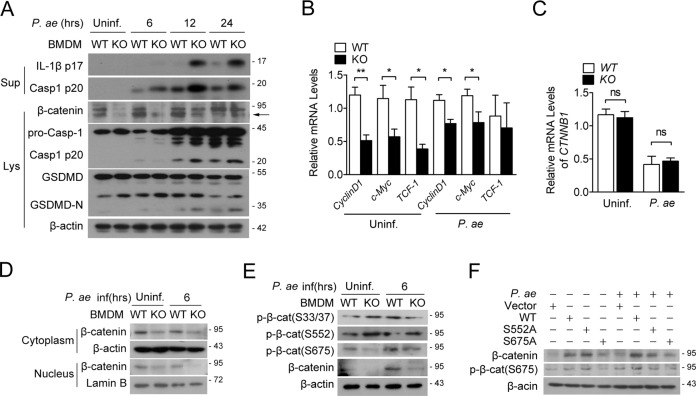
TREM2 promotes β-catenin stability and phosphorylation. **A**–**E** TREM2 KO vs. WT BMDMs were infected by *Pseudomonas aeruginosa* (MOI = 1) for indicated times. **F** RAW264.7 cells were transiently transfected with pcDNA3.1 vector or plasmids encoding WT or mutant (S552A or S675A) β-catenin, followed by *Pseudomonas aeruginosa* infection. **A**, **D**, **E**, **F** Protein levels of indicated molecules in cell lysates, cytoplasm, nucleus or supernatants of each group were detected by western blot. **B**, **C** mRNA levels of indicated genes were detected by real-time PCR. Data are presented as the mean ± SEM of three independent experiments. *P < 0.05; **P < 0.01. ns no significance.

Previous study reported that deficiency of TREM2 impairs β-catenin signaling during the process of osteoclast differentiation. Therefore, we next investigated whether β-catenin participate in TREM2-regulated pyogenic bacteria-induced macrophage pyroptosis. Interestingly, the protein abundance of β-catenin was decreased by TREM2 deficiency (Fig. 4A), which changed simultaneously with the increase of Caspase-1, IL-1β and GSDMD activation. In addition, loss of TREM2 decreased the transcription of *CyclinD1*, *c-Myc*, and *TCF-1*, which are main target genes of β-catenin signaling, while remained the same at β-catenin mRNA (*CTNNB1*) level, indicating that TREM2 modulated β-catenin at protein level rather than mRNA hierarchy (Fig. 4B, C). We next separated cytosolic and nuclear protein and found that TREM2 deficiency decreased β-catenin protein level in both cytoplasm and nucleus (Fig. 4D). Furthermore, we studied the effect of TREM2 on β-catenin phosphorylation and found that loss of TREM2 decreased the phosphorylation of β-catenin at Serine 675, rather than Serine 33/37 or Serine 552 (Fig. 4E). Transgene of a mutant β-catenin of S675A into macrophage was less able to maintain β-catenin protein level compared to WT or S552A mutant (Fig. 4F). These data together indicate that TREM2 stabilize β-catenin protein in both cytoplasm and nucleus by promotes β-catenin phosphorylation at Serine 675.

### TREM2 inhibits macrophage pyroptosis via β-catenin

**Fig. 5:**
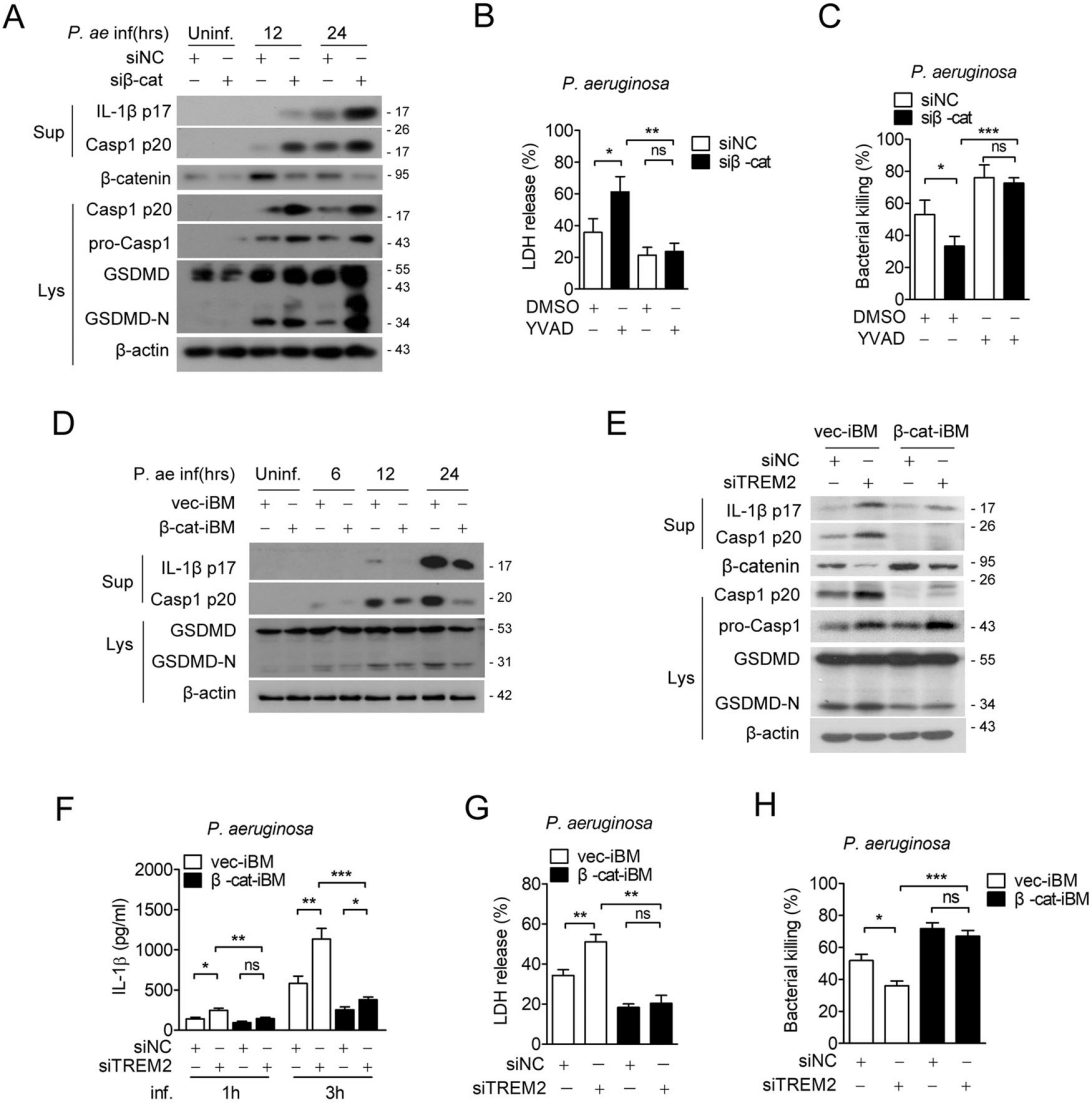
TREM2 inhibits *Pseudomonas aeruginosa*-induced pyroptosis via β-catenin. **A** Mouse BMDMs were transfected with siβ-catenin (siβ-cat) or siNC and then infected by *Pseudomonas aeruginosa*. **B**, **C** BMDMs were transfected with siβ-catenin vs. siNC, and then treated with Ac-YVAD-CMK (40 μM) or vehicle control (DMSO), followed by *Pseudomonas aeruginosa* infection. **D** vec-iBMs or β-cat-iBMs were infected with *Pseudomonas aeruginosa* for indicated time points. **E**–**H** vec-iBM and β-cat-iBM were transfected with siTREM2 vs. siNC, followed by *Pseudomonas aeruginosa* infection. **A**, **D**, **E** Protein levels of pro-Caspase-1, cleaved-Caspase-1, cleaved-IL-1β, and GSDMD in cell lysates or supernatants were tested by western blot. **B**, **G** Cytotoxicity was detected by LDH release assay. **C**, **H** Macrophage killing efficiency to *Pseudomonas aeruginosa* was assessed by the plate count method. **E** Secretion of IL-1β at 1 or 3 h post-infection was tested by ELISA. Data are presented as the mean ± SEM of three independent experiments. *P < 0.05; **P < 0.01; ***P < 0.001. ns no significance.

To study the role of β-catenin on macrophage pyroptosis and bacterial clearance, we knockdown β-catenin with a mixture of specific small interfering RNAs in macrophages followed by *Pseudomonas aeruginosa* infection. The western-blot results showed that knockdown of β-catenin enhanced the cleavage of Caspase-1, IL-1β and GSDMD at indicated time points after pyogenic bacterial infection (Fig. 5A). Consistently, the proportion of LDH release (Fig. 5B) and the fraction of PI^+^ cells (Supplementary Fig. 1A, B) were also increased in β-catenin knockdown cells, while this effect was abrogated by YVAD (Fig. 5B, Supplementary Fig. 1A, B). We further established a couple of transgenic iBMDM cell strains, vec-iBM and β-cat-iBM, which are stably expressing vector control or exogenous β-catenin respectively (Supplementary Fig. 1D). Inversely, the activation of pyroptosis biomarkers (Fig. 5D), the cell death ratio (Supplementary Fig. 1E, F) and the LDH release (Supplementary Fig. 1G) in β-cat-iBM were significantly lower than vec-iBM.

**Fig. 6:**
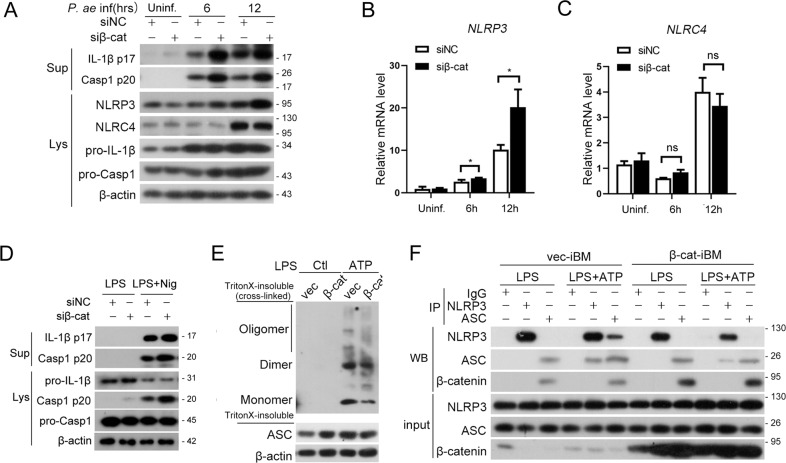
β-catenin suppresses NLRP3 inflammasome activation. **A**–**C** WT BMDMs were transiently transfected by siβ-catenin vs. siNC, and then infected by *Pseudomonas aeruginosa* (MOI = 1) for indicated times. **D** siβ-cat or siNC transfected BMDMs were treated with LPS or LPS plus Nigericin (10 μM) for 1 h. **E** vec-iBM or β-cat-iBM were stimulated with LPS (100 ng/ml, 3 h) or LPS plus ATP (4 mM, 1 h), and then cell lysates were detected by ASC oligomerization assay. **F** vec-iBM or β-cat-iBM were stimulated with LPS (100 ng/ml, 3 h) or LPS plus ATP (4 mM, 1 h), and then cells were collected and analyzed by co-immunoprecipitation using anti-NLRP3 or anti-ASC Ab or relative IgG isotypes. **A**, **D**, **E**, **F** Protein levels of indicated molecules in cell lysates or supernatants of each group were detected by western blot. **B** mRNA levels of NLRP3 and NLRC4 were detected by real-time PCR. Data are representative of at least three experiments. Error bars represent the mean ± SEM. *P < 0.05; **P < 0.01; ***P < 0.001, ns no significance.

Next, we investigate weather β-catenin involved in TREM2-mediated regulation on macrophage pyroptosis. Consistent with previous results, knockdown of TREM2 in vec-iBM promoted Caspase-1/IL-1β/GSDMD activation (Fig. 5E), IL-1β release (Fig. 5F), macrophage death (Supplementary Fig 1H, I) and LDH release (Fig. 5G). However, all of these changes in β-cat-iBM cells were compensated by the overexpressed β-catenin (Fig. 6E–G). These results indicate that β-catenin mediates the negative effect of TREM2 on macrophage pyroptosis.

Bacteria killing assay showed that β-catenin silence inhibited BMDMs killing to *Pseudomonas aeruginosa*, while treatment of YVAD counteracted this effect (Fig. 5C). In contrary, the bacterial killing of macrophages to pyogenic bacteria were enhanced in β-cat-iBM cells compared to vec-iBM cells. In addition, silencing of TREM2 also impaired bacterial killing in vec-iBM cells, while it was restored in β-cat-iBM cells (Fig. 5H). These data demonstrate that β-catenin is required for the TREM2-involved macrophage killing to pyogenic bacteria.

### β-catenin inhibits NLRP3 inflammasome activation by regulating its priming and assembly

As the previous results demonstrated that TREM2 prevents macrophages pyroptosis by regulating NLRP3 inflammasome, we furtherly studied the role of β-catenin on NLRP3 inflammasome activation. Consistent with the deficiency of TREM2, β-catenin knockdown also increased the expression of NLRP3 at both protein level (Fig. 6A) and mRNA level (Fig. 6B, C) while had no effect on that of NLRC4. Besides, knockdown of β-catenin accelerated Nigericin induced Caspase-1 and IL-1β cleavage (Fig. 6D). Next, we studied the effect of β-catenin on NLRP3 inflammasome assembly in vec-iBM and β-cat-iBM and found that overexpression of β-catenin decreased the LPS plus ATP triggered ASC oligomerization in macrophages (Fig. 6E). Furthermore, co-IP analysis showed that the LPS plus ATP induced NLRP3-ASC association also attenuated by β-catenin overexpression, indicating that β-catenin prevents NLRP3 inflammasome assembly (Fig. 6F). Interesting, the endogenous immunoprecipitation analysis showed that β-catenin could interact with ASC rather than NLRP3 with or without the stimulation of ATP (Fig. 6F), indicating that β-catenin probably target ASC to prevent NLRP3-ASC of ASC-ASC interaction. Taken together, these results revealed that β-catenin suppresses NLRP3 inflammasome activation and macrophage pyroptosis by inhibiting NLRP3 protein expression and NLRP3 inflammasome assembly.

## Discussion

As a PRR expressing on myeloid cell surface, TREM2 plays a critical role in regulating inflammatory responses and bacterial elimination [29–31]. Our previous study demonstrated that in *Pseudomonas aeruginosa* keratitis, deficiency of TREM2 elevated mouse corneas inflammation and bacterial burden by inhibiting macrophage pyroptosis [14, 16]. However, how TREM2 regulates macrophage pyroptosis and whether it affects macrophages clearance to other pyogenic bacteria were unknown. In the present study, we demonstrate that TREM2 inhibits pyogenic bacteria like *Staphylococcus aureus*, *Pseudomonas aeruginosa*, *Streptococcus pneumoniae*, and *Escherichia coli*-induced macrophage pyroptosis to promote bacterial eradication, which provides a novel mechanism of TREM2-mediated host defense against pyogenic bacteria.

To be noted, the present study demonstrated that blockage of pyroptosis by either inhibition or knockout of Caspase-1 or GSDMD enhanced the macrophage-mediated pyogenic bacterial clearance. This observation is consistent with the conclusion in other in vivo studies that knockout or inhibition of Caspase-1 enhances bacterial clearance in *Pseudomonas aeruginosa* keratitis [16, 32] or pneumonia [33]. Moreover, in vivo studies with respiratory infection of *Staphylococcus aureus* have demonstrated that neutralization of NLRP3 improves bacterial clearance, while blocking IL-1β/IL-18 does not [34]. Kambara et al. reported that GSDMD-deficiency augmented bactericidal activity to *Escherichia coli* in vivo by delaying neutrophil death [35]. These reports indicate a detrimental effect of macrophage pyroptosis on pyogenic bacterial eradication.

In contrast, Miao et al. demonstrated that macrophage pyroptosis protects host against *Salmonella, Legionella* and *Burkholderia* by releasing these intracellular bacteria into the extracellular space and exposing them to the uptake and killing by neutrophils [21]. It is also reported that macrophage pyroptosis triggers pore-induced intracellular traps (PITs, which is conceptually parallel to the neutrophil extracellular traps) to trap intracellular pathogens and then drives the recruitment of and efferocytosis by neutrophil to kill them [36]. Base on the literature, we speculate that the distinct roles of pyroptosis on host response to pyogenic bacteria vs. intracellular bacteria may due to their different abilities to evade immune defense. Pyogenic bacteria like *Pseudomonas aeruginosa* via inducing cells pyroptosis to reduce phagocytes numbers and to escape from intracellular killing. While intracellular pathogens like *Salmonella Typhimurium* are able to survive and replicate in monocytes/macrophages, and most of them may result in chronic or latent infection. Pyroptotic cell death of macrophages exposes the intracellular bacteria to other phagocytes, and therefore promotes the elimination of these intracellular pathogens in vivo.

We next explored the downstream molecules of TREM2 in modulating macrophage pyroptosis. Several studies suggested that β-catenin is required in maintaining cell viability and preventing cell death [37, 38]. TREM2 or its adaptor molecule DAP12 activates β-catenin in M-CSF-stimulated myeloid cells [25] or osteoclast precursors [24]. In the present study, we found that TREM2 deficiency suppressed bacteria-induced β-catenin accumulation in both cytoplasm and nuclear, and decreased β-catenin phosphorylation at Ser675 rather that Ser33/37 or Ser552. It is reported that phosphorylation at Ser33/37 or Ser552 promotes protein degradation or signal activation respectively, while Ser675 phosphorylation associates with signaling activation, protein stability and nuclear translocation [26]. Transfection of S675A β-catenin didn’t increase the protein levels of β-catenin as the WT or S552A isoform done, indicating the effect of Ser675 phosphorylation on β-catenin protein stabilization. Together, these data suggested that TREM2 induces β-catenin phosphorylation at Ser675 to promote β-catenin stabilization and accumulation in both nucleus and cytoplasm.

NLRP3 inflammasomes responses to many types of pyogenic bacterial infection [39**–**42] and plays important roles in different infectious disease such as respiratory infection[34], cystic fibrosis [43] and keratitis [44]. It also receives multiple modulatory signaling from other molecular to realize complex immunoregulation in anti-microbe response [45, 46]. Our data suggested TREM2/β-catenin pathway negatively regulated bacteria-induced NLRP3 canonical inflammasome activation. TREM2/β-catenin not only suppressed NLRP3 expression, but also inhibited ASC oligomerization and the ASC-NLRP3 association. On the one hand, deficiency of TREM2 or β-catenin decreased NLRP3 proteins and mRNAs level. We speculated that TREM2 promotes β-catenin accumulation in nucleus to suppress *NLRP3* transcription based on the observation that TREM2 increasing β-catenin nuclear location. On the other hand, CO-IP data showed that β-catenin interacted with ASC rather than NLRP3, what’s more, its binding to ASC decreased the interaction of ASC with NLRP3. Thus, we proposed that β-catenin may competitively inhibit NLRP3-ASC association, and therefore interfere with the assembly of NLRP3 inflammasome complex. NLRC4 is another important inflammasome responses to pyogenic bacterial infection, especially for *Pseudomonas aeruginosa* infection [33, 47]. However, we observed that TREM2/β-catenin have no effect on NLRC4 proteins and mRNAs level. Also, TREM2 deficiency did not influence NLRC4 interaction with ASC or Caspase-1. These data together suggests that TREM2/β-catenin specifically inhibits NLRP3 inflammasome at both primary and activation stages.

To be noted, Huang et al. reported that β-catenin promotes NLRP3 inflammasome activation in mouse peritoneal macrophages, by increasing NLRP3 inflammasome activation without affecting the priming stage [48]. They also used exogenous Co-IP in 293T cells to demonstrate that overexpression of β-catenin increases the association between NLRP3 and ASC. In contrast, other studies by Yue et al. and Li et al. both reported that β-catenin deficiency in BMDMs increased NLRP3 protein level and Caspase-1/IL-1β cleavage [49, 50]. In the present study, we examined the role of β-catenin on NLRP3 inflammasome activation in both primary and immortalized mouse BMDMs, and found that β-catenin inhibited the NLRP3 inflammasome activation at both priming and activation stage. Noteworthily, our endogenous Co-IP data demonstrated that β-catenin competed with NLRP3 for ASC binding, and thus inhibited NLRP3 inflammasome assembly and activation in BMDMs. Since β-catenin is localized in the cell membrane as well as cytoplasmic and nuclear compartments, each with distinct roles [26]. We speculate that this discrepancy of β-catenin-mediated NLRP3 regulation may due to distinct stimuli and cell types. To sum up, we found that TREM2/β-catenin suppressed pyogenic bacteria-induced macrophage pyroptosis by inhibiting NLRP3 inflammasome expression and activation, and therefore promoted macrophage-mediated bacteria elimination. These findings may provide new insight to understand the complex relationships among PRRs, host cell death, and bacterial elimination.

## Supplementary information

checklist

Original Data File

Original Data File

Original Data File

Original Data File

Original Data File

Supplementary Information

## Data Availability

All datasets generated and analyzed during this study are included in this published article and its Supplementary Information files. Additional data are available from the corresponding author on reasonable request.
