# Supplementary figures and images for: TREM2/β-catenin attenuates NLRP3 inflammasome-mediated macrophage pyroptosis to promote bacterial clearance of pyogenic bacteria
Source: Cell Death Dis. 2022 Sep 6;13(9):771. doi: 10.1038/s41419-022-05193-x (PMC9448748; doi:10.1038/s41419-022-05193-x)

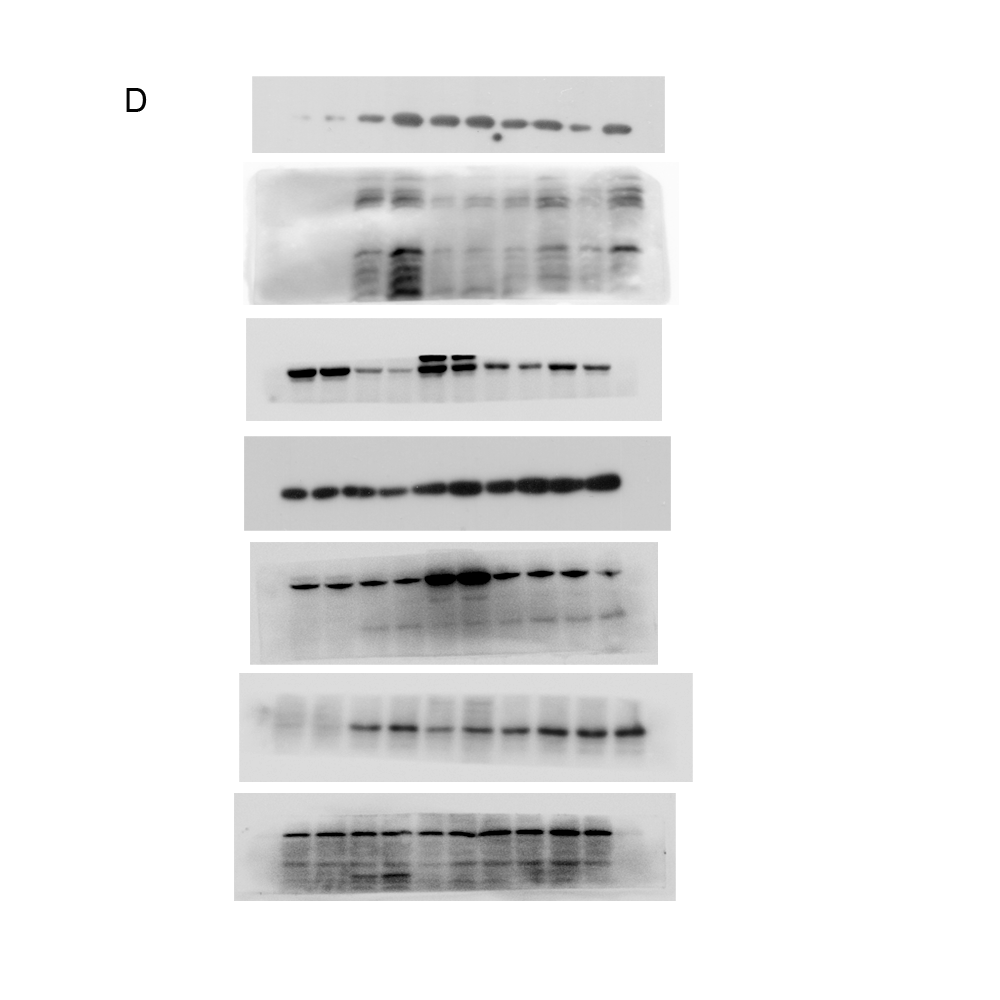

Supplement: Supplementary file 2 — Original Data File [file 41419_2022_5193_MOESM2_ESM.tif]

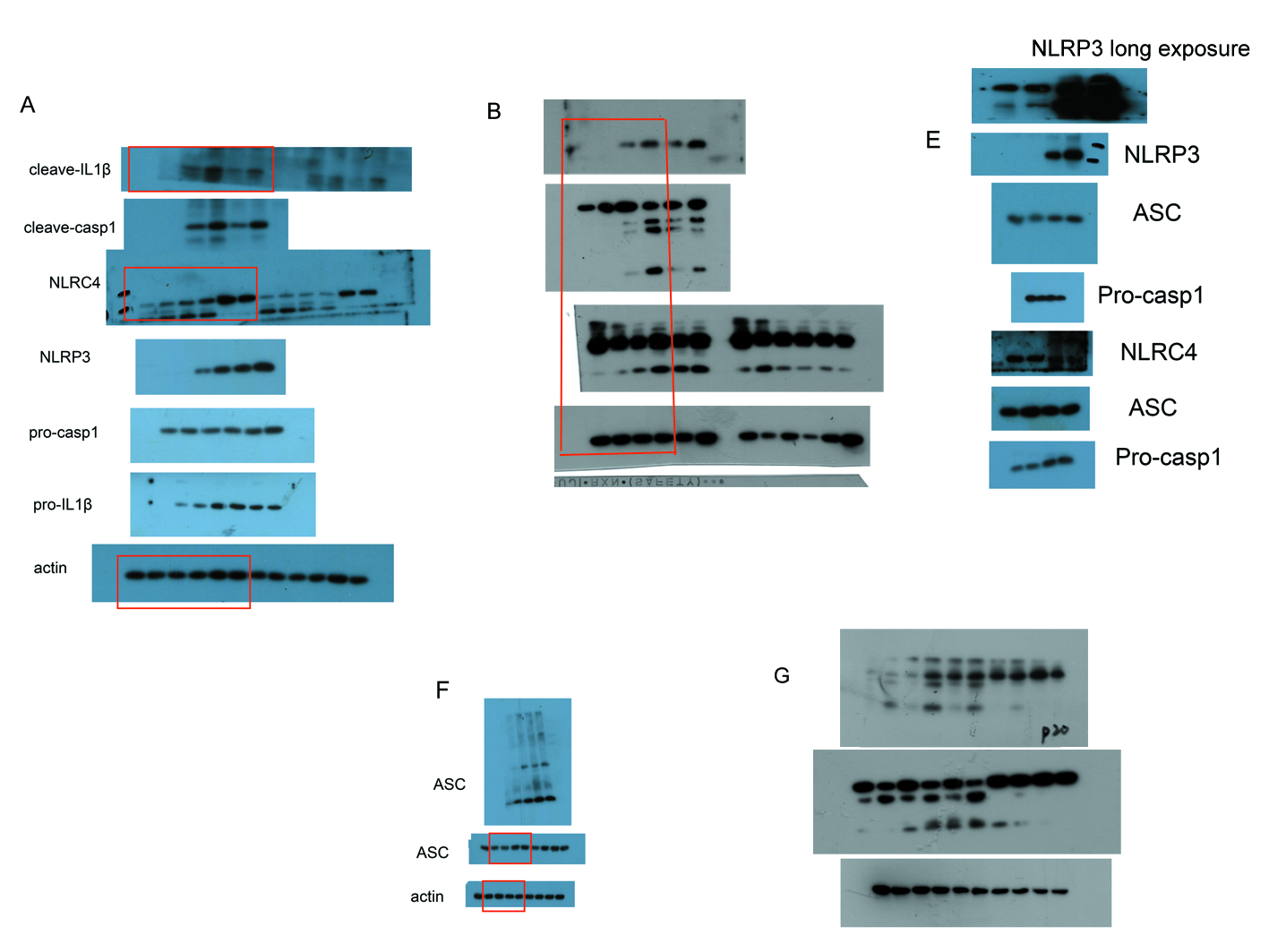

Supplement: Supplementary file 3 — Original Data File [file 41419_2022_5193_MOESM3_ESM.tif]

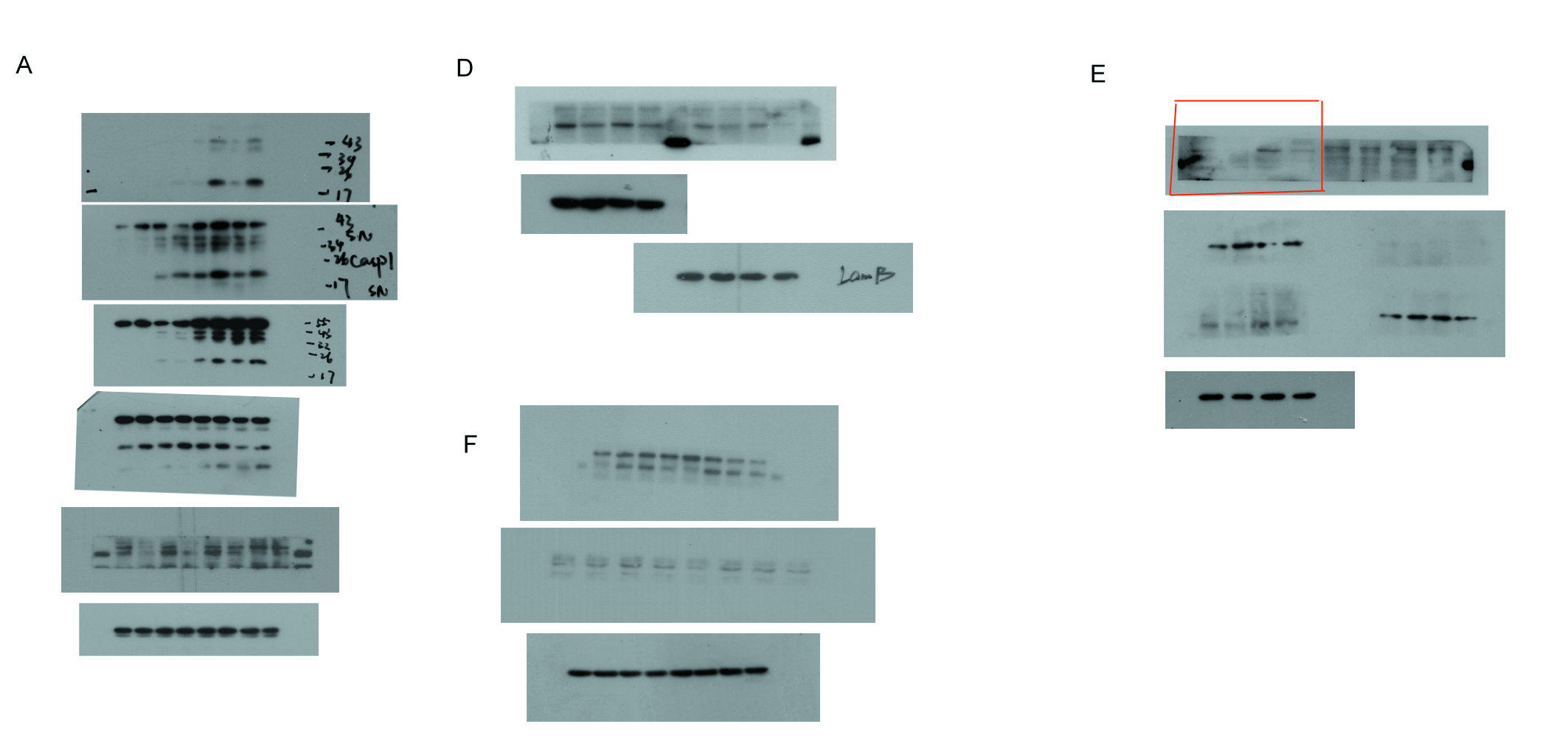

Supplement: Supplementary file 4 — Original Data File [file 41419_2022_5193_MOESM4_ESM.tif]

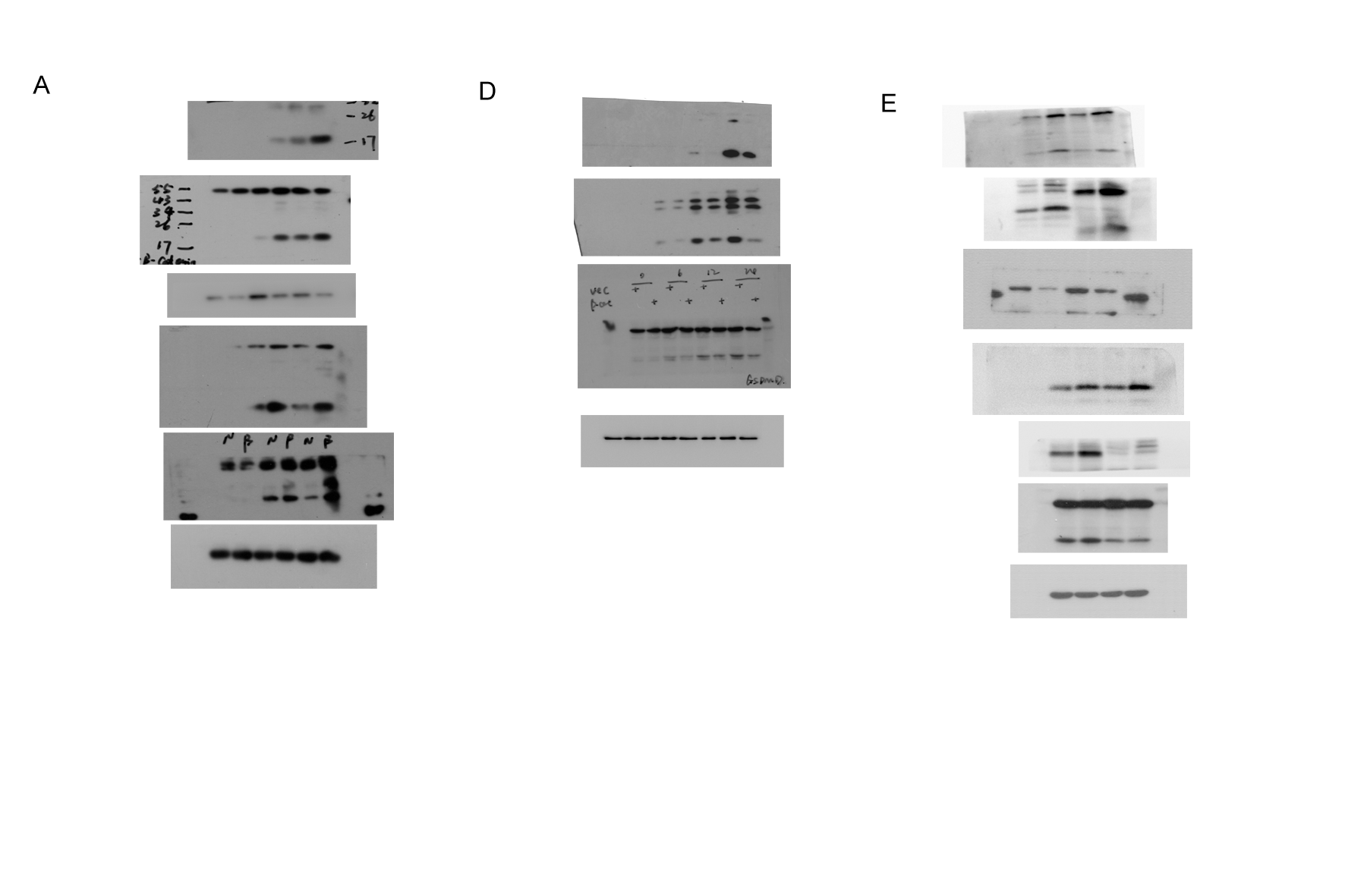

Supplement: Supplementary file 5 — Original Data File [file 41419_2022_5193_MOESM5_ESM.tif]

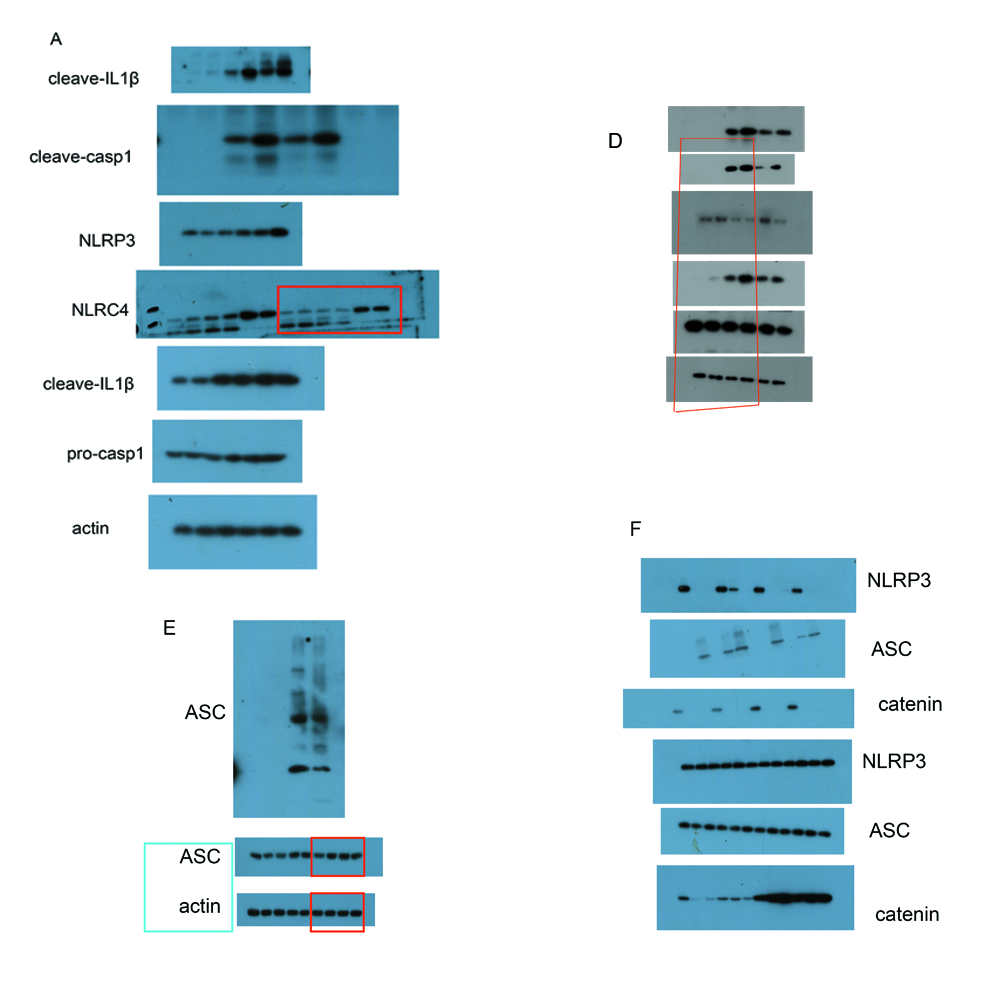

Supplement: Supplementary file 6 — Original Data File [file 41419_2022_5193_MOESM6_ESM.tif]
