# Supplementary material for: TREM2/β-catenin attenuates NLRP3 inflammasome-mediated macrophage pyroptosis to promote bacterial clearance of pyogenic bacteria
Source: Cell Death Dis. 2022 Sep 6;13(9):771. doi: 10.1038/s41419-022-05193-x (PMC9448748; doi:10.1038/s41419-022-05193-x)

**Supplementary Information**

**Supplementary Table 1. Source and catalog information of antibodies and reagents.**

| **REAGENT or RESOURCE** | **SOURCE** | **IDENTIFIER** |
| --- | --- | --- |
| **Antibodies** | | |
| NLRP3 | AdipoGen | AG-20B-0014-C100 |
| caspase-1 | AdipoGen | AG-20B-0042 |
| NLRC4 | AdipoGen | G-CAB13117.50 |
| IL-1β | R&D | AF-401-NA |
| ASC(D2W8U) | CST | #67824 |
| β-Catenin | CST | 8480 |
| Phospho-β-Catenin (Ser33/37) | CST | 2009 |
| Phospho-β-Catenin (Ser552) (D8E11) | CST | 5651 |
| Phospho-β-Catenin (Ser675) | CST | 9567 |
| Phospho-Akt (Ser473) (D9E) | CST | 4060 |
| Phospho-GSK-3β (Ser9) | CST | 9336 |
| Lamin B1 | Santa Cruz | sc-374015 |
| β-Actin | Sigma-Aldrich | SAB3500350 |
| TREM2 | abcam | ab209841 |
|  |  |  |
| **Chemicals, Peptides, and Recombinant Proteins** | | |
| LPS | sigma | L4391-1MG |
| ATP | Invivogen | tlrl-atp |
| Nigericin | Invivogen | tlrl-nig |
| Flagellin | Invivogen | tlrl-pafla |
| Ac-YVAD-CMK | Invivogen | inh-yvad |

**Supplementary Figure 1. TREM2 inhibits *Pseudomonas aeruginosa*-induced macrophage death via β-catenin.** (A, B) BMDMs were transfected with siβ-catenin vs siNC, and then treated with Ac-YVAD-CMK (40 μM) or vehicle control (DMSO), followed by *Pseudomonas aeruginosa* infection. (C) Vec-iBMs or β-cat-iBMs were infected with infected by *Pseudomonas aeruginosa*, *Staphylococcus aureus*, *Streptococcus pneumoniae* and *Escherichia coli* (MOI=20). (D-G) vec-iBMs or β-cat-iBMs were infected with *Pseudomonas aeruginosa* for indicated MOIs. (H, I) vec-iBM and β-cat-iBM were transfected with siTREM2 vs siNC, followed by *Pseudomonas aeruginosa* infection. (A, E, H) Cell death was analyzed by flow cytometry with PI staining. (B, F, I) Quantification of flow cytometry analysis of dead cells (PI^+^). (C) The bacterial killing efficiency was assessed by bacterial killing assay based on plate count. (D) Protein levels of β-catenin of each group were detected by western blot. (G) Cytotoxicity was detected by LDH release assay. **P*<0.05. ***P*<0.01. ns, no significance.


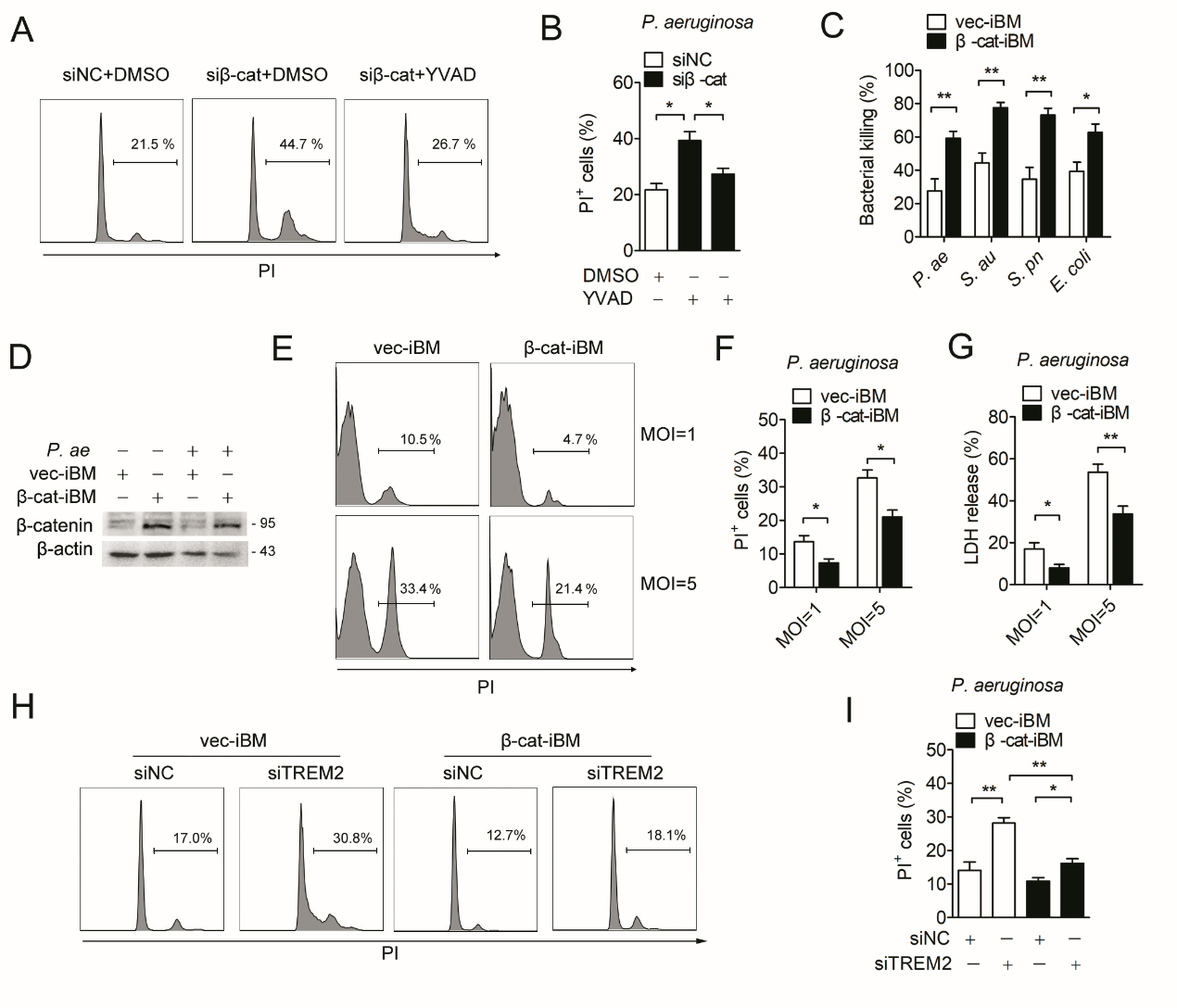

Supplement: Supplementary file 7 — Supplementary Information [file 41419_2022_5193_MOESM7_ESM.docx]
